# Modaline sulfate promotes Oct4 expression and maintains self-renewal and pluripotency of stem cells through JAK/STAT3 and Wnt signaling pathways

**DOI:** 10.1186/s13578-021-00669-3

**Published:** 2021-08-04

**Authors:** Xianglin Mei, Hanhan Zhao, Huihan Ai, Shuyue Wang, Zhenbo Song, Lihua Zheng, Guannan Wang, Ying Sun, Yongli Bao

**Affiliations:** 1 National Engineering Laboratory for Druggable Gene and Protein Screening, Northeast Normal University, Changchun, 130117 China; 2 NMPA Key Laboratory for Quality Control of Cell and Gene Therapy Medicine Products, Northeast Normal University, Changchun, 130024 China

**Keywords:** Modaline sulfate, Oct-4, Stem cell, Wnt signaling pathways, JAK/STAT3 signaling pathways

## Abstract

**Background:**

Stem cells have been extensively explored for a variety of regenerative medical applications and they play an important role in clinical treatment of many diseases. However, the limited amount of stem cells and their tendency to undergo spontaneous differentiation upon extended propagation in vitro restrict their practical application. Octamer-binding transcription factor-4 (Oct4), a transcription factor belongs to the POU transcription factor family Class V, is fundamental for maintaining self-renewal ability and pluripotency of stem cells.

**Methods:**

In the present study, we used the previously constructed luciferase reporters driven by the promoter and 3’-UTR of Oct4 respectively to screen potential activators of Oct4. Colony formation assay, sphere-forming ability assay, alkaline phosphatase (AP) activity assay and teratoma-formation assay were used to assess the role of modaline sulfate (MDLS) in promoting self-renewal and reinforcing pluripotency of P19 cells. Immunofluorescence, RT-PCR, and western blotting were used to measure expression changes of stem-related genes and activation of related signaling pathways.

**Results:**

We screened 480 commercially available small-molecule compounds and discovered that MDLS greatly promoted the expression of Oct4 at both mRNA and protein levels. Moreover, MDLS significantly promoted the self-renewal capacity of P19 cells. Also, we observed that the expression of pluripotency markers and alkaline phosphatase (AP) increased significantly in MDLS-treated colonies. Furthermore, MDLS could promote teratoma formation and enhanced differentiation potential of P19 cells in vivo. In addition, we found that in the presence of LIF, MDLS could replace feeder cells to maintain the undifferentiated state of OG2-mES cells (Oct4-GFP reporter gene mouse embryonic stem cell line), and the MDLS-expanded OG2-mES cells showed an elevated expression levels of pluripotency markers in vitro. Finally, we found that MDLS promoted Oct4 expression by activating JAK/STAT3 and classic Wnt signaling pathways, and these effects were reversed by treatment with inhibitors of corresponding signaling pathways.

**Conclusions:**

These findings demonstrated, for the first time, that MDLS could maintain self-renewal and pluripotency of stem cells.

**Supplementary Information:**

The online version contains supplementary material available at 10.1186/s13578-021-00669-3.

## Background

Stem cells such as embryonic stem cells (ESCs) share the significant property of self-renewal and pluripotency [1]. Because of these properties, stem cells are being investigated for a variety of regenerative medical applications and treatments of many diseases, such as diabetes mellitus (DM) and systemic lupus erythematosus (SLE) [2–4]. Excitingly, some of these trials result in significant impact [5]. However, one of the bottlenecks in the stem cell therapy is that stem cells tend to differentiate into other types of cells during culture in vitro. Therefore, finding a way to effectively maintain the pluripotency of stem cells cultured in vitro is important for the application of stem cells.

Pluripotency has been known to be controlled through an extensive transcriptional network such as transcription factors, cytokines and external signals [6, 7]. Among the core pluripotency-associated transcriptional regulators, Octamer-binding transcription factor-4 (Oct4) [8], SRY-related high-mobility-group -box protein-2 (Sox2) [9], Cellular myelocytomatosis oncogene (c-Myc) [10], and Nanog [11, 12] is central to the machinery governing pluripotency [13]. Oct4, also known as Oct3 and Oct3/4, was originally shown to be essential in regulating self-renewal and pluripotency properties in ESCs [8]. It is expressed in ESCs, embryonic germ (EG) cells and embryonic carcinoma (EC) cells and essential for early embryonic development [14, 15]. For example, in the absence of Oct4, embryos will die at the time of implantation for the reason that pluripotent inner cell mass cells ^[[14]]^ and ESCs couldn’t differentiate into trophectoderm [8, 16]. Moreover, recent studies indicated that Oct4 was essential in cellular reprogramming [17]. Accordingly, Oct4 is the most significant target for inducing reprogramming and maintaining pluripotency.

In recent years, several lines of evidence have suggested that small-molecule compounds could been used to maintain the pluripotency of ESCs [1, 18, 19]. Retinol, the alcohol form of Vitamin A, could up-regulate the expression of Nanog and prevented the differentiation of mouse ESCs [20]. In addition, Retinol could overcome the requirement for feeder cells in ESCs and prevent differentiation via yet unknown mechanism [21]. It has been discovered that SC1 could maintain the self-renewal of mES cells by inhibiting Ras GAP- and ERK1-dependent signaling pathways in the absence of feeder cells, leukemia inhibitory factor and serum [22]. Ethyl-p-methoxycinnamate also had the ability to enhance Oct4 expression and reinforce pluripotency of mES cells through the NF-κB signaling pathway [23].

In the present study, using the previously constructed luciferase reporters driven by the promoter and 3’-UTR of Oct4, we screened the small-molecule compounds that could maintain cell self-renewal and pluripotency. Our results suggested that modaline sulfonate (MDLS) could significantly promote the expression of Oct4, and it had a potential role in maintaining the stemness of P19 cells. MDLS could also replace feeder cells to maintain the undifferentiated state of OG2-MESC in the presence of LIF. The mechanistic studies demonstrated that MDLS up-regulated the expression of Oct4 and reinforced pluripotency at least partially through the activation of JAK / STAT3 and classic Wnt signaling pathways. Overall, the studies described here demonstrated a new function of MDLS in maintaining self-renewal and pluripotency.

## Methods

### Cell lines and cell culture

P19 cells (mouse embryonic carcinoma cells line), Oct4-GFP reporter gene mouse embryonic stem cells (OG2-mES) and HEK-293 T cells (human embryonic kidney cell line) were obtained from the Shanghai Institute for Biological Sciences Cell Resource Center, Chinese Academy of Sciences. Mouse embryonic fibroblast (MEF) cells were prepared in the laboratory. P19 cells were cultured in MEMα medium (Gibco-BRL, Gaithersburg, MD, USA), supplemented with 10% fetal bovine serum (FBS; TBD Science, Tianjin, China). For the differentiation studies, P19 cells were cultured in MEMα, supplemented with 10% FBS in the presence of 500 nM of retinoic acid (RA; Sigma, St. Louis, MO, USA). HEK-293 T cells and MEF cells were maintained on Dulbecco’s modified Eagle’s medium (DMEM; Corning, New York, USA), supplemented with 10% FBS. OG2-mES cells were cultured in mitomycin C (Invitrogen, Carlsbad, CA, USA)-treated MEFs in knockout DMEM (Invitrogen, Carlsbad, CA, USA), which was supplemented with 15% knockout serum replacement (Invitrogen, Carlsbad, CA, USA), 2 mM L-glutamine (Invitrogen, Carlsbad, CA, USA), 1 × nonessential amino acids (Invitrogen, Carlsbad, CA, USA), 1 × 10^3^ units/mL LIF (Merck Millipore, Billerica, MA, USA) and 0.1 mM 2-mercaptoethanol (Invitrogen, Carlsbad, CA, USA). All the cells were cultured at 37℃ with 5% CO_2_.

### Antibodies and reagent

The monoclonal antibody against Oct4 and β-catenin, the polyclonal antibodies against Sox2, were obtained from Santa Cruz Biotechnology (CA, USA). The rabbit polyclonal antibodies against Nanog, AKT, p-AKT, STAT3, p-STAT3 and class III β-tubulin were purchased from Cell Signaling Technology (Beverly, MA, USA). The rabbit polyclonal antibodies against alpha-fetoprotein (AFP) and a mouse monoclonal antibody against cardiac troponin (cTnT) were purchased from Abcam (Cambridge, MA, USA). The mouse monoclonal antibody against GAPDH (glyceraldehyde-3-phosphate dehydrogenase) was obtained from Kangcheng Biotech (Shanghai, China). 3-(4, 5-Dimethylthiazol-2-yl)-2, 5-diphenyltetra zolium bromide (MTT) was purchased from Sigma Chemical Co. (St. Louis, MO, USA). Fluorescein isothiocyanate-conjugated goat anti-mouse immunoglobulin G (IgG) antibody, Cyanine3 (Cy3)-conjugated goat anti-rabbit IgG antibody, Cyanine3 (Cy3)-conjugated goat anti-mouse IgG antibody, enhanced chemiluminescence (ECL)-Plus kit, Alkaline Phosphatase (AP) Detection Kit and 4,6-diamidino-2-phenylindole (DAPI) were purchased from Beyotime Institute of Biotechnology (Shanghai, China). All the small-molecule compounds used in the study were purchased from Taosu Biotech (Shanghai, China), and their purity was greater than 95%. All the small-molecule compounds were dissolved in dimethyl sulfoxide (DMSO, Sigma-Aldrich, St. Louis,MO, USA) as a 10 mg/mL stock solution. β-Catenin inhibitor salinomycin and JAK1 inhibitor ruxolitinib were purchased from MedChemExpress (Beijing, China).

### Plasmid preparation and transfection

The pGL3-basic reporter plasmid and pGL3-Oct4 plasmid were previously constructed in our laboratory [23]. The plasmids pEZX-MT01 and pEZX-MT01-Oct4 mRNA 3'UTR were purchased from GeneCopoeia (Guangzhou, China). Briefly, HEK-293 T cells were plated onto each well of a 24-well plate for 24 h before transfection. Then, HEK-293 T cells were transfected with pGL3-Oct4p (pEZX-Oct4 mRNA 3'UTR) and pGL3-basic (pEZX-MT01) using Entranster-D (Engreen, Beijing, China) following the manufacturer’s protocols. P19 cells were transfected with pGL3-Oct4p (pEZX-Oct4 mRNA 3'UTR) and pGL3-basic (pEZX-MT01), plus with pCMV-β-galactosidase plasmids using Lipofectamine 2000 (Invitrogen, Carlsbad, CA, USA) according to the manufacturer’s instructions.

### Compound screening

In the primary screening, HEK-293T cells were plated onto a six-well plate at 1 × 10^5^ cells/well. After 24 h, the cells were transfected with pGL3-Oct4p or pEZX-MT01-Oct4 mRNA 3'UTR reporter using Entranster-D and maintained in DMEM. After 4 h, the transfected cells were replated onto 96-well plates at a density of 8 × 10^3^ cells/well. After 24 h, the cells were treated with compounds at final concentrations of 5 μg/mL in DMEM with 3% FBS for 24 h. The luciferase activity was assayed described in a previous study [24].

In the secondary screening, P19 cells were plated onto a 24-well plate at a density of 4 × 10^4^ cells/well. After 24 h, the cells were transfected with pGL3-Oct4p (pEZX-Oct4 mRNA 3'UTR) plasmids and pGL3-basic (pEZX-MT01) vector plasmids per well plus pCMV-β-galactosidase plasmids using Lipofectamine 2000. After 24 h, the cells were treated with the identified compounds at a final concentration of 5 μg/mL or DMSO (the negative control). After 44 h of incubation, the luciferase activity of the cells was assayed and normalized to β-galactosidase activity using the FLUOstar OPTIMA system (BMG Labtech, Offenburg, Baden-Wuerttemberg, Germany).

### RNA extraction and RT-qPCR

Total RNA was prepared from the cells using TRIzol reagent (Invitrogen, Carlsbad, CA, USA) following the manufacturer’s protocols. Subsequently, the cDNA was synthesized with a reverse transcription System (Promega, Madison, WI, USA) according to manufacturer’s instructions. RT-qPCR was completed using the SYBR ExScript RT-qPCR kit (Takara Biotechnology Co., Ltd.). The primers of Oct4 were as follows: 5′-CTGCAGAAGGAGCTAGAACAGTTTG-3′ (sense) and 5′-GATGGTGGTCTGGCTGAACACCTTTCC-3′ (anti-sense); and GAPDH, 5′-GCCAAAAGGGTCATCATCTC-3′ (sense) and 5′-GTAGAGGCAGGGATGATGTTC-3′ (anti-sense). The PCR program was as follows: Denaturation at 95℃ for 10 min; followed by 40 cycles at 95℃ for 10 s, 60℃ for 1 min. The mRNA levels were quantified using the 2^−∆∆Cq^ method and normalized to GAPDH.

### Western blot analysis

Cells were harvested and the proteins were extracted. Western blot analysis was performed as previously described [25]. Briefly, proteins were resolved on SDS-PAGE, transferred onto polyvinylidenedifluoride transfer membranes (PVDF), and blocked with 5% non-fat dry milk in TBST buffer for 1 h at room temperature. The membranes were then incubated with the diluted primary antibodies overnight at 4℃, washed three times, followed by the incubation with HRP-conjugated secondary antibodies for 1 h at room temperature. The bands were detected with an EZ-ECL kit (BI Biological Industries, 20-500-120) in the MicroChemi bio-imaging system (DNR).

### Cell viability assay

The cells were plated in triplicate in 96-well plates. 12 h later, the cells were treated with various concentrations (see figure legend) of the indicated compounds for 44 h. Cell viability was measured by the MTT assay. The cell viability was determined according to the following equation: Cell viability = OD value of experimental group/OD value of control group × 100%.

### Plate colony-forming assay and Alkaline Phosphatase Assay

P19 cells were placed onto 6-well plates at a density of 1000 cells/well, and gently shaken to make them evenly distributed in the plate without aggregation. Then, the fresh culture medium containing the compounds was replaced every day. After one week, the cells were fixed with 4% paraformaldehyde (PFA) for 15 min at room temperature. Subsequently, the fixed cells were washed twice with PBS and then stained using the Alkaline Phosphatase Detection Kit (Beyotime Biotechnology, China) following protocols provided by the manufacturer.

### Suspension culture

A six-well plate was coated with 1 mL of 0.5% agarose per well in advance to perform suspension culture. After washing the plate with PBS, P19 cells were plated onto the plate at a density of 1 × 10^4^ cells/well. Then, the cells were added with the final concentration of 5 μg/mL compounds or DMSO. The culture medium was refreshed every other day. The plates were maintained at 37 ℃ for 7 days before the colonies were examined.

### Immunofluorescence assay

Immunofluorescence staining was performed as previous described [26]. Briefly, the cells were fixed with 4% paraformaldehyde and permeabilized with 0.25% Triton X100. After washing with PBS, the cells were incubated with 5% bovine serum albumin in PBS for 1 h at room temperature for blocking nonspecific binding protein. Then, the cells were labeled with primary antibodies against Nanog and Oct-4 overnight. After washing with PBS, the cells were incubated with a fluorochrome-conjugated secondary antibody. The cells were analyzed by the Olympus BX50 fluorescence microscope (Olympus, Tokyo, Japan).

### Teratoma formation assay

Female Balb/c nude mice 6–8-weeks old were used for the study (Vital River Laboratory Animal Technology Company, Beijing, China). All the animal experiments were conducted in accordance with the established guidelines. The Chinese Academy of Sciences Animal Care and Use Committee gave approval for the animal experiments. The P19 cells were treated with MDLS or DMSO for 7 days. Subsequently, the P19 cells were harvested at a density of 5 × 10^7^ cells/mL. Then, 200 μL of the cell suspension was injected subcutaneously into nude mice. After 3 weeks, the mice were euthanized by the dislocated cervical vertebrae and the teratomas were resected and measured using calipers.

### Hematoxylin–eosin staining

The teratoma specimens were fixed in 10% neutral-buffered formalin (Solarbio, Beijing, China) and embedded in paraffin (Solarbio, Beijing, China). The representative sections were stained routinely with hematoxylin (Solarbio, Beijing, China) and eosin (Solarbio, Beijing, China) and examined under a light microscope [26].

### Self-renewal assay of OG2-mES cells in the presence of MDLS

Undifferentiated OG2-mES cells were plated onto gelatin-coated black 96-well plates at a density of 1 × 10^4^ cells/well without feeder cells. After 24 h, the medium was replaced by fresh medium with or without LIF but containing 5 μg/mL MDLS or DMSO. After another 6 days of incubation, in which the media and MDLS were changed on day 3, the cells were analyzed for morphology and GFP fluorescence intensity.

### Expansion of OG2-mES cells in the presence of MDLS

OG2-mES cells were plated onto gelatin-coated six-well plates at a density of 1 × 10^4^ cells/well and cultured with 5 μg/mL MDLS, BMP4 or DMSO without feeder cells. They were split every 3 days and seeded at the same density. The OG2-mES cells at passage 2, 3, 4 and 5 were harvested and analyzed by western blot or fluorescence-activated cell sorting (FACS).

### Statistical analysis

All data collected in this study were obtained from at least three independent experiments. We used the SPSS, Excel and FlowJO software to analyze these data. An analysis of variance (ANOVA) was carried out by two-tailed Student’s t-test. The significance level was set to *P < 0.05, **P < 0.01 and ***P < 0.001, unless stated otherwise. Error bars denoted the standard deviation (SD).

## Results

### Screening of potential activators of Oct4 expression

**Fig. 1:**
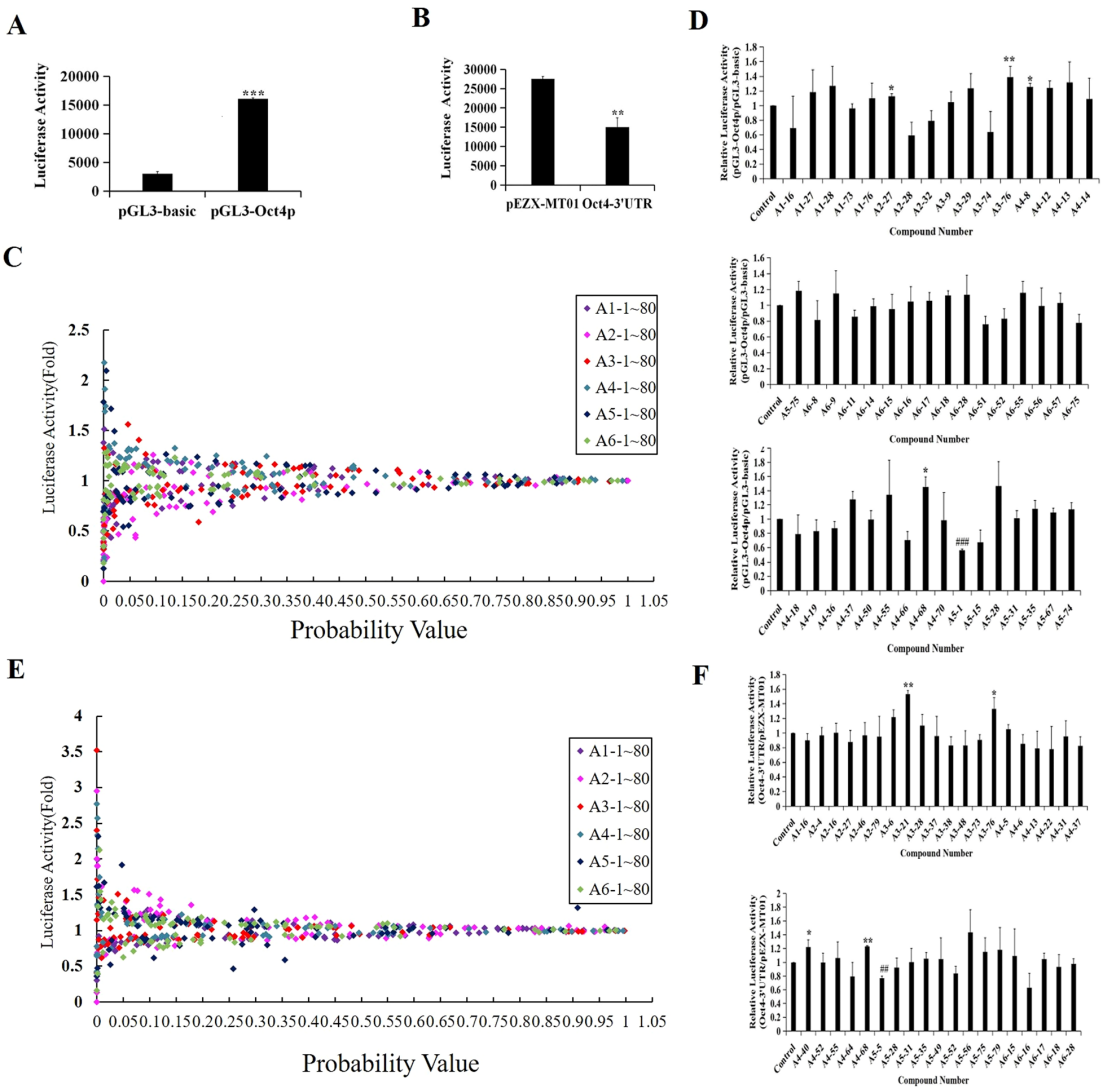
Screening of potential activators of Oct4. **A** The activities of the Oct4 promoter-driven firefly luciferase reporter. HEK-293 T cells were transfected with the pGL3-Oct4 and pGL3-basic reporter plasmid, plus with pCMV-β-galactosidase plasmids. 48 h later, the cells were lysated and the luciferase activities was detected. **B** The activities of the pEZX-MT01-Oct4 mRNA 3'UTR reporter plasmid. HEK-293 T cells were transfected with pEZX-MT01 and pEZX-MT01-Oct4 mRNA 3'UTR plasmids, plus with pCMV-β-galactosidase plasmids. 48 h later, the cells were lysated and the luciferase activities was detected. **C** Primary screening using Oct4 promoter-driven firefly luciferase reporter. HEK-293 T cells were transfected with the pGL3-Oct4p plasmid for 24 h and then treated with small-molecule compounds (5 μg/mL) for 24 h. DMSO was used as a negative control. Subsequently, the luciferase activity was measured. The results were expressed as the fold induction (over the activity of the negative control). **D** Secondary screening for compounds obtained from (**C**). P19 cells were co-transfected with pGL3-Oct4p or pGL3-basic vector and pCMV-β-galactosidase plasmid for 24 h and then treated with the identified compounds (5 μg/mL) for 24 h. DMSO was used as a negative control. Subsequently, the luciferase activity was measured and normalized to β-galactosidase activity. The results were expressed as the fold induction (over the activity of the negative control). **E** Primary screening using Oct4 mRNA 3'UTR reporter plasmid. HEK-293T cells were transfected with a pEZX-Oct4 mRNA 3'UTR plasmid for 24 h and then treated with 480 small-molecule compounds (5 μg/mL) for 24 h. DMSO was used as a negative control. Subsequently, the luciferase activity was measured. The results were expressed as the fold induction (over the activity of the negative control). **F** Secondary screening for compounds obtained from (**E**). P19 cells were co-transfected with pEZX-Oct4 mRNA 3'UTR or pEZX-MT01 vector and pCMV-β-galactosidase plasmid for 24 h and then treated with the identified compounds (5 μg/mL) for 24 h. DMSO was used as a negative control. Subsequently, the luciferase activity was measured and normalized to β-galactosidase activity. The results were expressed as the fold induction (over the activity of the negative control). *P < 0.05, **P < 0.01, ***P < 0.001, compared with the negative control

Oct4 is essential in the establishment and maintenance of pluripotency [27]. To identify compounds that selectively target Oct4, we first confirmed the activities of the previously constructed luciferase reporters driven by the promoter and 3’-UTR of target gene Oct4. The result revealed that the luciferase activity of the Oct4 promoter-driven firefly luciferase reporter up-regulated and the luciferase activity of the Oct4 mRNA 3'UTR-driven firefly luciferase decreased compared with control reporter (Fig. 1A and B) [23, 28], indicating that the above screening systems can be used for the subsequent screening of compounds.

Subsequently, 480 small-molecule compounds were screened in HEK-293T cells. As showed in Fig. 1C, we found 48 compounds could significantly enhance the Oct4 promoter activity. Then, these 48 compounds were examined by the secondary screening assay in P19 cells, and we found that four compounds (A2-27, A-3-76, A4-8 and A4-68) could significantly enhance the Oct4 promoter activity in P19 cells (Fig. 1D). Furthermore, these small-molecule compounds were also screened using the Oct4 mRNA 3'UTR- driven luciferase reporter. As showed in Fig. 1E and F, four compounds (A3-21, A3-76, A4-40 and A4-68) could target Oct4 mRNA 3'UTR.

**Fig. 2:**
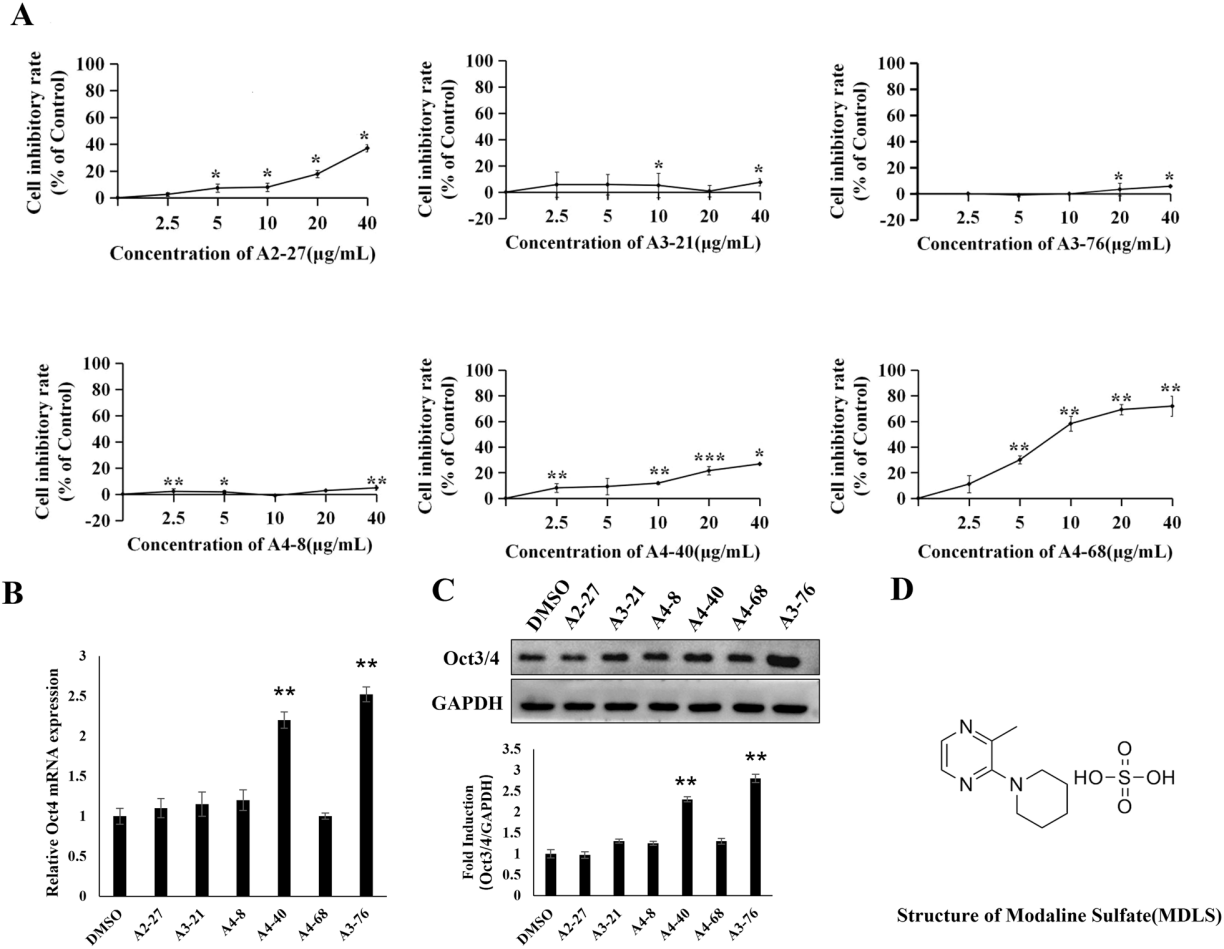
The effect of the potential compounds on the expression of Oct4. **A** Cytotoxicity of the potential compounds against P19 cells. The P19 cells were treated with increasing concentrations of the potential compounds (2.5, 5, 10, 20 and 40 μg/mL) for 44 h. Cell viability was measured by the MTT assay. **B** The effect of MDLS on the expression of Oct4 at the mRNA level. P19 cells were treated with MDLS or DMSO for 24 and Oct4 mRNA levels were analyzed using RT-qPCR. **C** The effect of MDLS on the expression of Oct4 at the protein level. Top: P19 cells were treated with the potential compounds or DMSO for 48 h. The cells were then lysed, and the expression of Oct4 was analyzed using the western blot analysis. Equal protein loading was evaluated using GAPDH. Bottom: quantitative analysis of Western blotting data by image J soft-ware. Levels of these proteins were normalized to the corresponding GAPDH band expressed as relative fold changes in comparison to control samples. Values correspond to the mean ± S.E. of three independent experiments. **D** Structure of modaline sulfate (MDLS). *P < 0.05, **P < 0.01, ***P < 0.001, compared with the negative control

To determine the non-toxic dose ranges of these six compounds, we tested their effects on viability of P19 cells. Data from Fig. 2A suggested that treatment of P19 cells with low concentrations of A3-76 almost did not affect cell proliferation and had no obvious cytotoxicity on P19 cells compared to other compounds. Most importantly, the potential role of these compounds in promoting Oct4 expression were investigated in P19 cells, and we observed that A3-76 had the most significant effect on Oct4 expression at both mRNA and protein level compared with other compounds(Fig. 2B and C). Therefore, we choose A3-76 (modaline sulfate; see structure in Fig. 2D) which presented the best *in vitro* cytotoxic results, as our leading compound for further investigations.

### The role of MDLS in the self-renewal of P19 cells

**Fig. 3:**
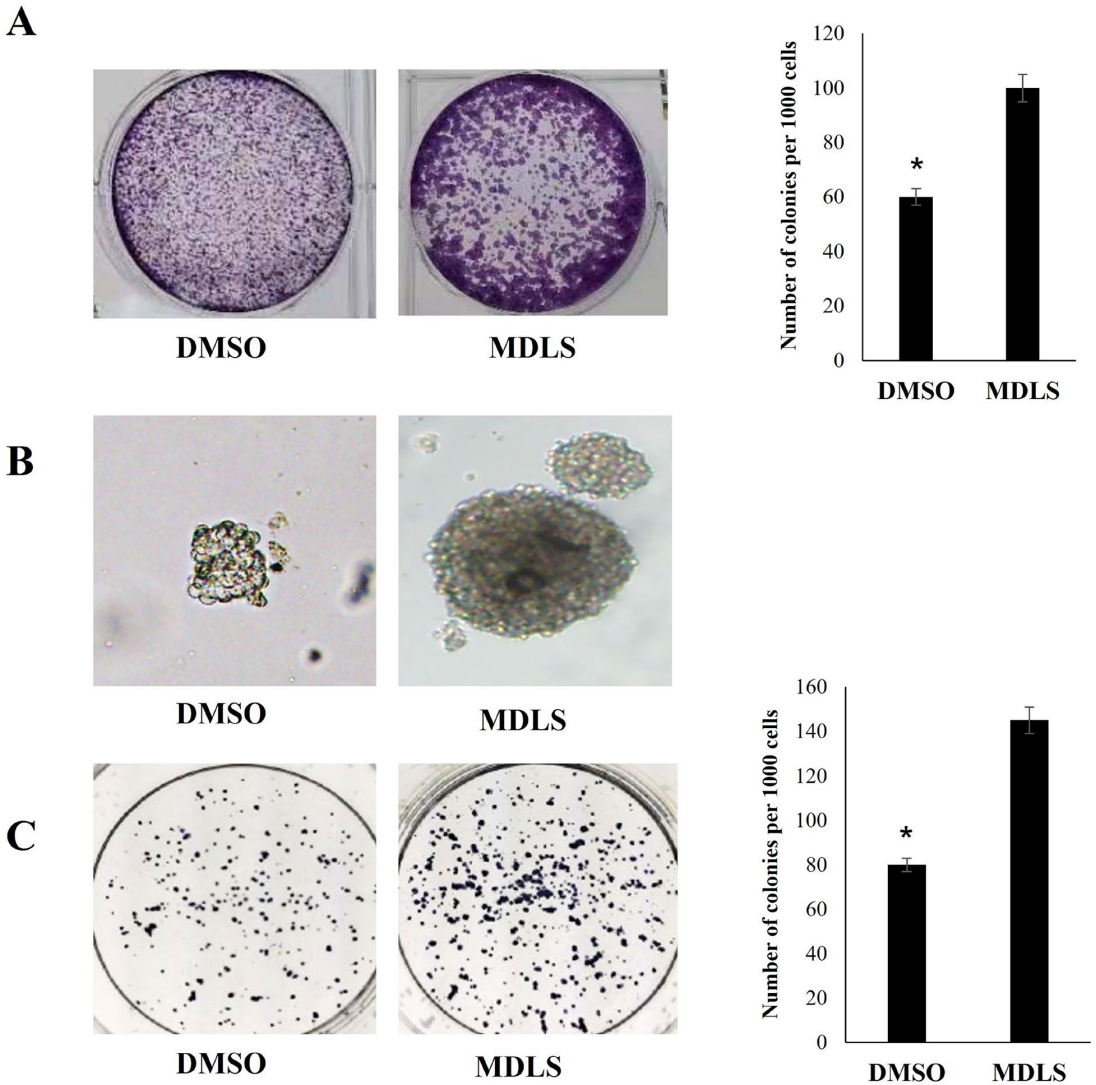
The role of the potential compounds in the self-renewal of P19 cells. **A** Effect of MDLS on the plate colony formation of P19 cells. P19 cells were plated onto 6-well plates for 24 h and then the culture medium was replaced with the fresh culture medium containing 5 μg/mL MDLS daily. After one week, the cells were stained with MTT and the colony formation was determined by the microscopic examination. **B** Effect of MDLS on the colony formation of P19 cells in hanging drop culture. P19 cells were made into a single-cell suspension and then cell suspension was added with 5 μg/mL of MDLS or DMSO. After that, the cells were counted and prepared at a density of 1 × 10^4^ cells/mL. Then, cells were placed on the underside of a petri dish lid in a 25 μl drop. The lid was inverted and placed on a plate containing 3 mL PBS. The morphological changes of the cells were observed under the microscope after 48 h. **C** Effect of MDLS on the colony formation of P19 cells in suspension culture. P19 cells were plated onto 0.5% agarose-coated six-well plates and treated with MDLS (5 μg/mL) or DMSO for 5 days. Subsequently, we harvested the embryoid bodies and transferred them to another 6-well plate. After adhering to the wall, the number of EBs was counted and the cells were stained with MTT

The ability for self-renewal is a key characteristic of stem cells, and colony-forming assay and sphere-forming ability assay are the common methods to test the self-renewal ability of stem cells [29]. As shown in Fig. 3A, MDLS-treated P19 cells could form more and bigger colonies in comparison with the control group in adherent culture. To further verify the effects of MDLS, we used suspension culture to test the colony formation efficiency. The results indicated that the typical embryoid bodies (EBs) could be formed in P19 cell after 48 h of treatment with MDLS (Fig. 3B). Moreover, the EBs could be maintained in suspension culture for up to 4 days (Fig. 3C). Since MDLS had little effect on cell proliferation (Fig. 2A), we inferred that MDLS promoted colony formation not by increasing cell proliferation. Overall, these results demonstrated that MDLS could reinforce the self-renewal capacity of P19 cells.

### MDLS reinforced the pluripotency of P19 cells

**Fig. 4:**
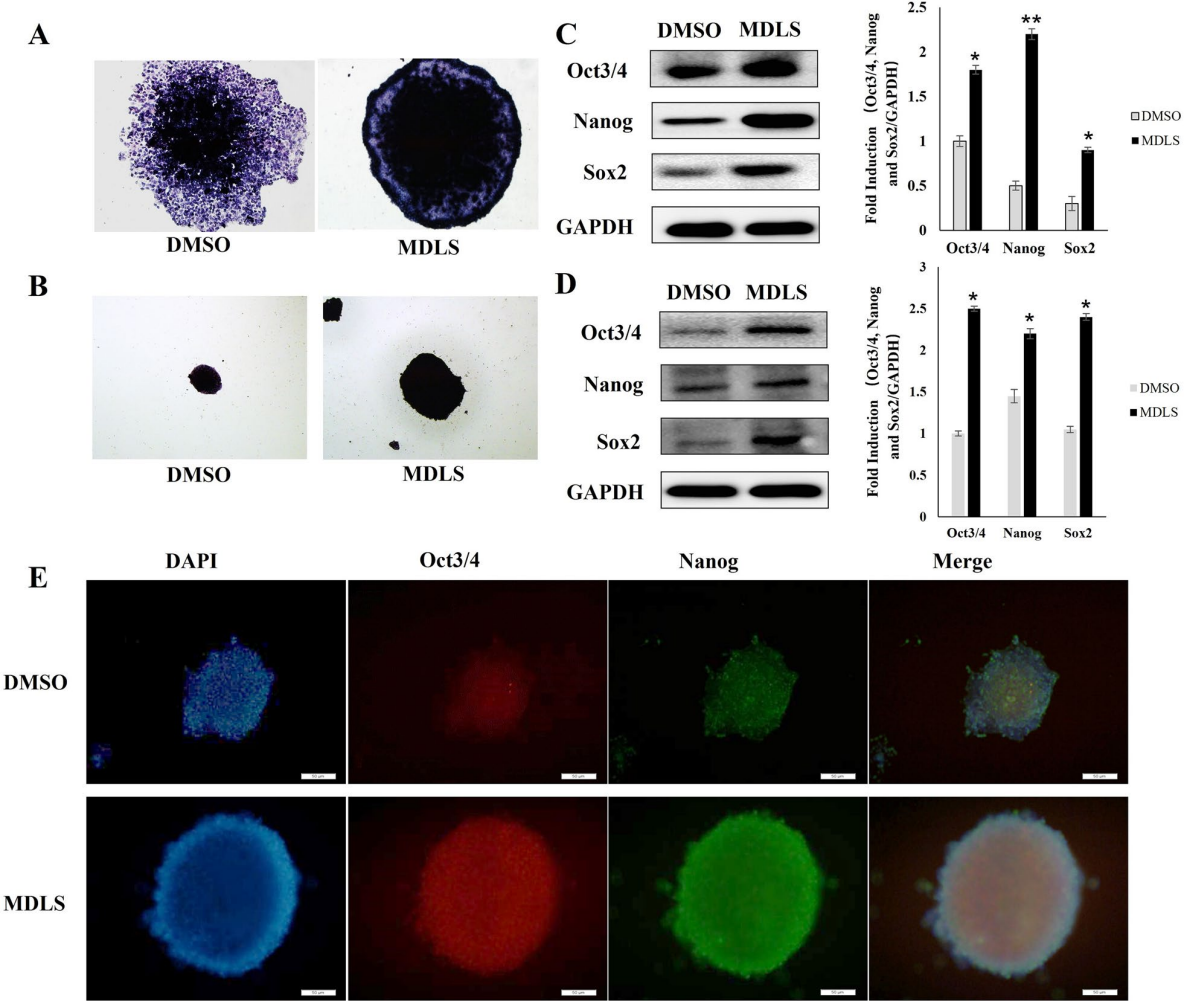
MDLS reinforced the pluripotency of P19 cells. **A** The alkaline phosphatase activity of MDLS-induced colonies in adherent culture. **B** The alkaline phosphatase activity of MDLS-induced EBs in suspension culture. **C** The expression of Oct4, Sox2, and Nanog (the pluripotency markers) in MDLS-induced colonies in adherent culture. Left: P19 cells were treated with MDLS (5 μg/mL) or DMSO for 48 h. Subsequently, the expression of Oct4, Sox2, and Nanog in the colonies was detected using Western blot analysis. Right: quantitative analysis of Western blotting data by image J soft-ware. Levels of these proteins were normalized to the corresponding GAPDH band expressed as relative fold changes in comparison to control samples. Values correspond to the mean ± S.E. of three independent experiments. **D** The expression of Oct4, Sox2, and Nanog (the pluripotency markers) in MDLS-induced EBs detected by western blot analysis. Left: P19 cells were plated onto 0.5% agarose-coated six-well plates and treated with MDLS (5 μg/mL) or DMSO for 5 days. The expression levels of Oct4, Sox2, and Nanog were detected by western blot analysis in embryoid bodies. Right: quantitative analysis of Western blotting data by image J soft-ware. Levels of these proteins were normalized to the corresponding GAPDH band expressed as relative fold changes in comparison to control samples. Values correspond to the mean ± S.E. of three independent experiments. **E** The expression of Oct4 and Nanog (the pluripotency markers) in MDLS-induced EBs detected by immunofluorescence assay

Based on the results above, we further investigated whether MDLS could reinforce the pluripotency of P19 cells. Alkaline phosphatase (AP) activity assay [30, 31], pluripotency markers analysis and the differentiation ability of teratomas were performed [32]. Firstly, we detected the effects of MDLS on the AP activity of MDLS-induced colonies and MDLS-induced EBs. Compared with the control group, MDLS-treated colonies showed almost 100% colonies that were phase bright with sharp boundaries and stained strongly positive for AP (Fig. 4A), indicating that MDLS could promote self-renewal ability of P19 cells. Similar results were observed in MDLS-induced EBs (Fig. 4B).

Furthermore, the expression of Oct4, Sox2, and Nanog (the pluripotency markers) in MDLS-induced colonies was investigated. The Weston blot analysis results showed that Oct4, Sox2, and Nanog were expressed at higher levels in P19 cells treated by MDLS (Fig. 4C) and MDLS-induced EBs (Fig. 4D) compared with the DMSO-treated groups. The immunofluorescence analysis also revealed that the expression levels of Oct4 and Nanog were higher in MDLS-induced EBs compared with the control group (Fig. 4E). These results suggested that MDLS could enhance the pluripotency of P19 cells.

**Fig. 5:**
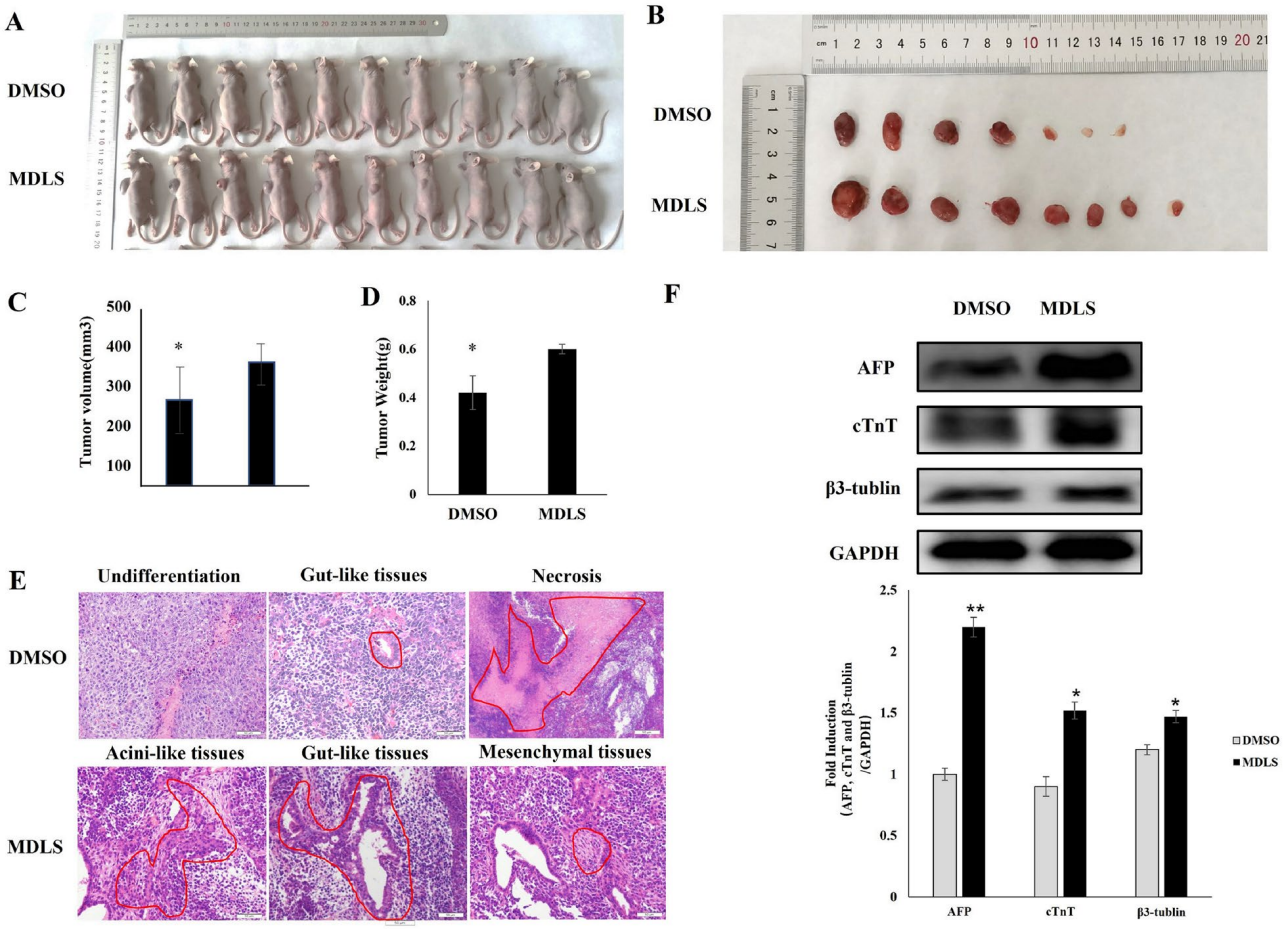
MDLS promoted teratoma formation of P19 cells and enhanced the differentiation potential of P19 cells. The P19 cells were treated with MDLS or DMSO for 7 days. Subsequently, the P19 cells were harvested at a density of 5 × 10^7^ cells/mL. Then, 200 μl of the cell suspension was injected subcutaneously into nude mice. After 3 weeks, the mice were euthanized by cervical dislocation, and the teratomas were resected and measured using calipers. **A** Phenotype of nude mice after 3 weeks of treatment. **B** Appearance of teratomas derived from MDLS-treated and DMSO-treated cells. **C** The volume of teratomas monitored at the end of the experiment. **D** The weight of teratomas monitored at the end of the experiment. **E** The paraffin sections from the teratoma tissue specimens were stained with hematoxylin and eosin, and these sections were observed under a light microscope. **F** Top: the expression levels of class III β-tubulin, cTnT and AFP in the teratomas of P19 cells were analyzed using Western blot analysis. Equal protein loading was evaluated using GAPDH. *P* < 0.05, compared with the DMSO-treated group. Bottom: quantitative analysis of Western blotting data by image J soft-ware. Levels of these proteins were normalized to the corresponding GAPDH band expressed as relative fold changes in comparison to control samples. Values correspond to the mean ± S.E. of three independent experiments

To further validate the results above, we employed a teratoma-formation assay to examine the role of MDLS in vivo. We observed that MDLS-induced P19 cells could form more and larger teratomas compared with the control group (Fig. 5A–D), indicating that MDLS reinforced the teratoma formation ability of P19 cells. Moreover, teratoma section stained with hematoxylin/eosin showed that the MDLS-treated teratomas were able to differentiate into more typical gut-like tissues and pancreatic acini-like tissues (endoderm), and mesenchymal tissues (mesoderm) can also be observed. Whereas only a few gut-like tissues (endoderm) with diffuse necrosis were observed in the control group (Fig. 5E). Furthermore, west blotting results showed that the expression levels of the markers of ectoderm (class III β-tubulin), mesoderm (cTnT) and endoderm (AFP) were remarkably up-regulated in MDLS-induced teratomas (Fig. 5F), suggesting that MDLS could enhance the pluripotency of P19 cells. Overall, these results confirmed that MDLS could significantly reinforce the pluripotency of P19 cells.

### MDLS enhanced the self-renewal of mESCs and promoted the expression of pluripotency markers in the expansion of mESCs

**Fig. 6:**
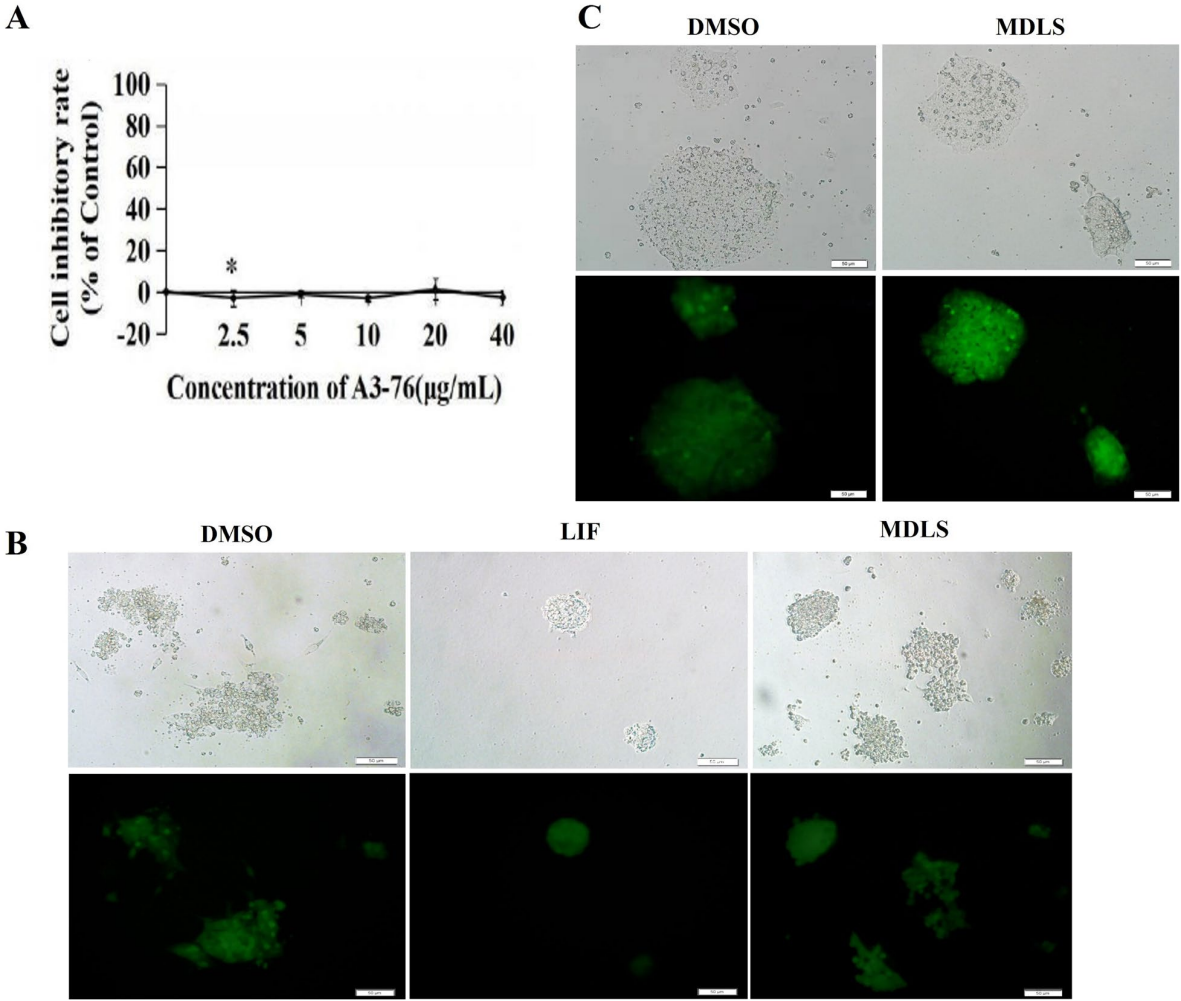
MDLS enhanced the self-renewal of mESCs. **A** The cytotoxic effect of MDLS on OG2-mES cells. OG2-mES cells were treated with different concentrations (2.5, 5, 10, 20, and 40 μg/mL) of MDLS for 44 h and MTT assay was conducted. The results were shown as mean ± SD of three separate experiments. **B** The role of MDLS in the self-renewal of OG2-mES cells in the absence of feeder cells and LIF. Undifferentiated OG2-mES cells were plated onto gelatin-coated black 96-well plates at a density of 1 × 10^4^ cells/well without feeder cells. After 24 h, the medium was replaced by fresh medium with or without LIF but containing 5 μg/mL MDLS or DMSO. After another 6 days of incubation, self-renewal of mESCs was examined by analyzing GFP fluorescence intensity. **C** The role of MDLS in the self-renewal of OG2-mES cells in the presence of LIF under feeder-free conditions

To investigate the role of MDLS in maintaining the stemness of mESCs, an established reporter mouse embryonic stem cell line OG2-mES, derived from heterozygous Oct4-GFP (with an 18-bp Oct4 regulatory region) transgenic OG2 mice was used [33]. Firstly, we estimated the cytotoxic effect of MDLS on OG2-mES cells using the MTT assay and the results showed that different concentrations (2.5, 5, 10, 20, and 40 μg/mL) of MDLS displayed almost no toxicity on OG2-mES cells. Finally, MDLS was used at a concentration of 5 μg/mL (Fig. 6A).

Typically, mES cells are maintained in culture with feeder cells and/or mixtures of exogenous factors. When cultured without feeder cells and LIF, OG2-mES cells would lose both GFP expression and their compact-colony morphology completely in 4–6 days, suggesting a decrease of self-renewal ability [22]. Therefore, we first explored whether MDLS could rescue the differentiation of OG2-mES in the absence of feeder cells and LIF. As shown in Fig. 6B, the OG2-mES cells cultured with MDLS for six days lost compact-colony morphology and GFP expression. These results suggested that MDLS alone was not sufficient to replace feeder cells and LIF to maintain the self-renewal of mESCs.

Subsequently, we investigated whether MDLS could promote self-renewal of mES cells in presence of LIF under feeder-free conditions. As shown in Fig. 6C, when OG2-mES treated with DMSO tended to differentiate obviously, the cells treated with MDLS could maintain normal colony morphology and the expression of GFP. These results suggested that MDLS could partially replace feeder cells to maintain the self-renewal of stem cells in presence of LIF.

**Fig. 7:**
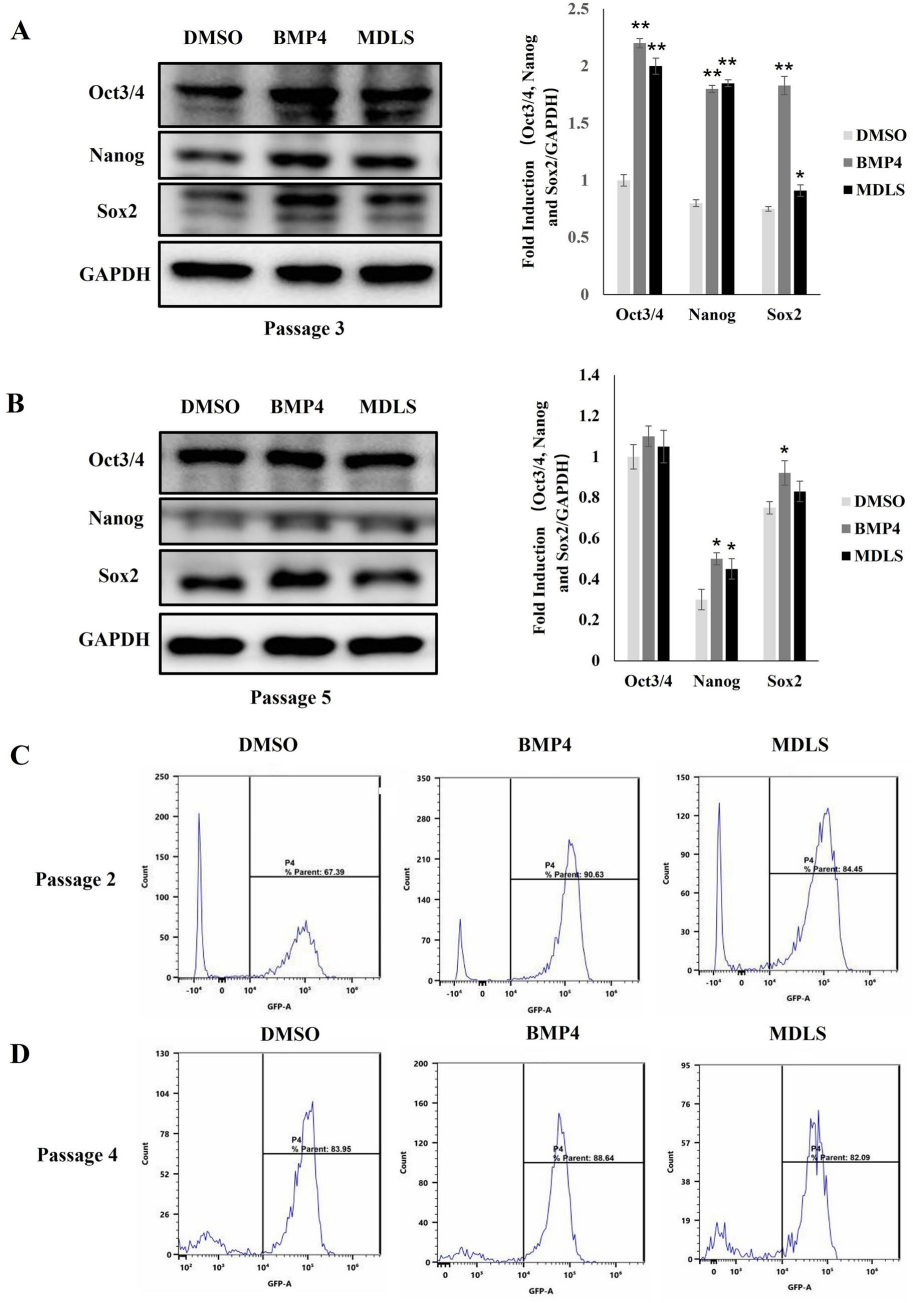
MDLS promoted the expression of pluripotency markers in the expansion of mESCs. OG2-mES cells were plated onto gelatin-coated six-well plates at a density of 1 × 10^4^ cells/well and cultured with 5 μg/mL MDLS, BMP4 or DMSO without feeder cells. They were split every 3 days and seeded at the same density. **A** Left: the expression of Oct4, Sox2 and Nanog in the OG2-mES cells treated by MDLS at passage 3 was detected using Western blot analysis. Right: quantitative analysis of Western blotting data by image J soft-ware. Levels of these proteins were normalized to the corresponding GAPDH band expressed as relative fold changes in comparison to control samples. Values correspond to the mean ± S.E. of three independent experiments. **B** Left: the expression of Oct4, Sox2and Nanog in the OG2-mES cells treated by MDLS at passage 5 was detected using Western blot analysis. Right: quantitative analysis of Western blotting data by image J soft-ware. Levels of these proteins were normalized to the corresponding GAPDH band expressed as relative fold changes in comparison to control samples. Values correspond to the mean ± S.E. of three independent experiments. **C** The proportion of Oct4-positive cells at passage 2 was analyzed with FACS. **D** The proportion of Oct4-positive cells at passage 4 was analyzed with FACS

To further elucidate the role of MDLS in the expansion of mESC in vitro, we used ESC-SR medium supplemented with MDLS and LIF to culture OG2-mES cells under feeder-free conditions, and the OG2-mES cells were analyzed for colony morphology and GFP fluorescence intensity, and BMP4 was used as the positive control. Normally, OG2-mES cells could only expand in vitro for five passages due to the lack of feeder cells [34]. Our results suggested that MDLS could significantly promote the expression of pluripotent markers (Oct4, Nanog and Sox2) in the third passage of OG2-mES cells (Fig. 7A), but the effect was weakened in the fifth passage (Fig. 7B). Moreover, Oct4-positive OG2-mES cells in passage 2 and passage 4 were measured by FACS analysis and we found that MDLS significantly increased the proportion of Oct4-positive cells in the second passage of OG2-mES cells (Fig. 7C), but there were no significant differences in the fourth passage of OG2-mES cells (Fig. 7D). These results indicated that MDLS could promote the expression of pluripotent markers and maintain the undifferentiated state at the early stage of OG2-mES cells expansion under feeder-free conditions.

### MDLS promoted the expression of Oct4 through the JAK/STAT3 and classic Wnt signaling pathway

**Fig. 8:**
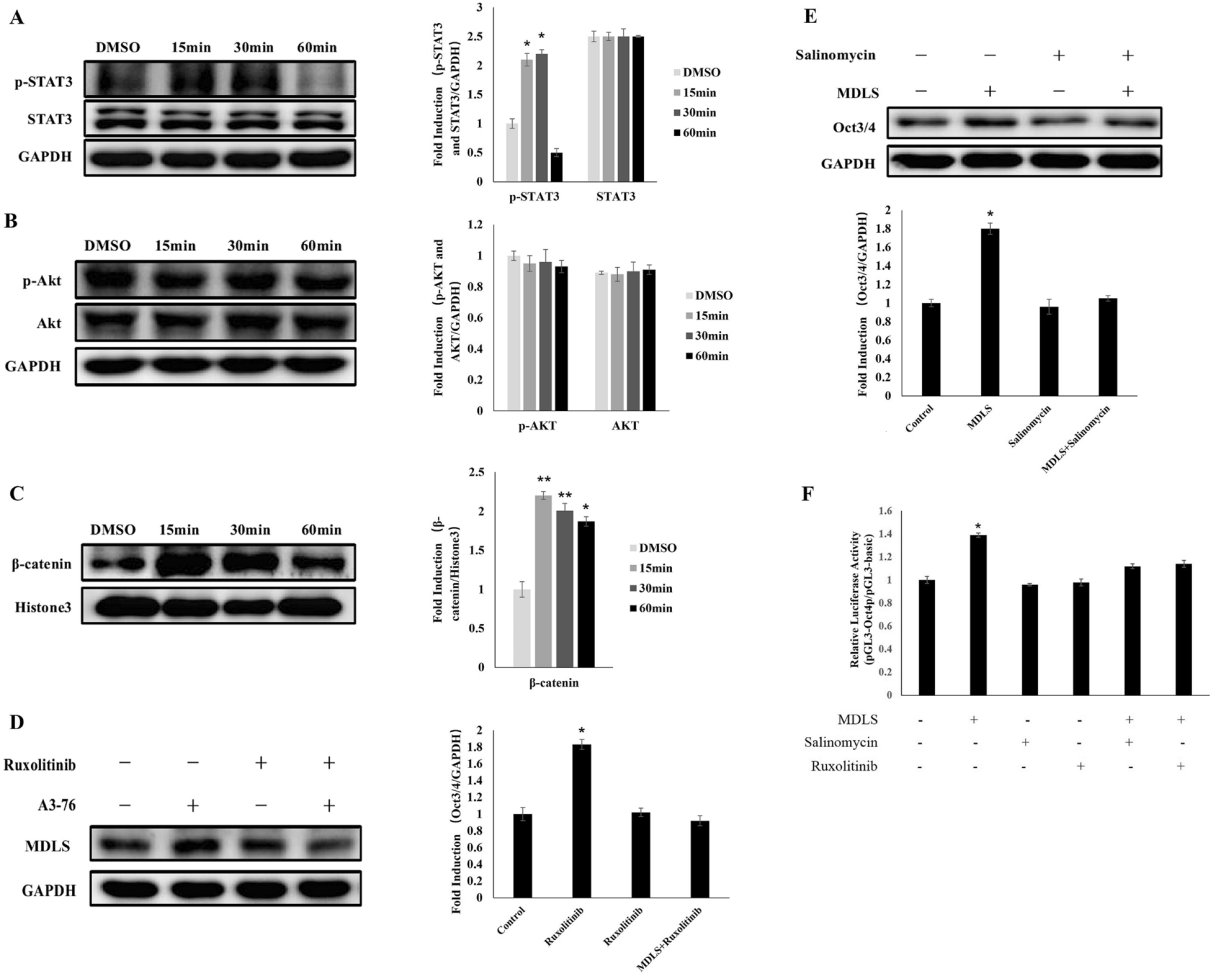
MDLS promoted the expression of Oct4 through the JAK/STAT3 and classic Wnt signaling pathway. **A** Left: the phosphorylation levels of STAT3 were detected by western blot analysis in P19 cells treated with MDLS for the indicated times. Right: quantitative analysis of Western blotting data by image J soft-ware. Levels of these proteins were normalized to the corresponding GAPDH band expressed as relative fold changes in comparison to control samples. Values correspond to the mean ± S.E. of three independent experiments. **B** Left: the phosphorylation levels of Akt were detected by western blot analysis in P19 cells treated with MDLS for the indicated times. Right: quantitative analysis of Western blotting data by image J soft-ware. Levels of these proteins were normalized to the corresponding GAPDH band expressed as relative fold changes in comparison to control samples. Values correspond to the mean ± S.E. of three independent experiments. **C** Left: the levels of β-Catenin in the nucleus were detected by western blot analysis in P19 cells treated with MDLS for the indicated times. Right: quantitative analysis of Western blotting data by image J soft-ware. Levels of these proteins were normalized to the corresponding Histone3 band expressed as relative fold changes in comparison to control samples. Values correspond to the mean ± S.E. of three independent experiments. **D** Ruxolitinib (JAK1 inhibitor) reversed the MDLS-induced up-regulation of Oct4. Left: P19 cells were treated with Ruxolitinib (2.5 μg/mL) and MDLS (5 μg/mL) or DMSO for 24 h. The expression of Oct4 was determined using Western blot analysis. Right: quantitative analysis of Western blotting data by image J soft-ware. Levels of these proteins were normalized to the corresponding GAPDH band expressed as relative fold changes in comparison to control samples. Values correspond to the mean ± S.E. of three independent experiments. **E** Salinomycin (β-Catenin inhibitor) reversed the MDLS-induced up-regulation of Oct4. Top: P19 cells were treated with Salinomycin (0.0078125 μg/ml) and MDLS (5 μg/mL) or DMSO for 24 h. The expression of Oct4 was determined using Western blot analysis. Bottom: quantitative analysis of Western blotting data by image J soft-ware. Levels of these proteins were normalized to the corresponding GAPDH band expressed as relative fold changes in comparison to control samples. Values correspond to the mean ± S.E. of three independent experiments. **F** P19 cells were co-transfected with pGL3-Oct4p or pGL3-basic vector and pCMV-β-galactosidase plasmid and treated with Salinomycin, Ruxolitinib and MDLS for 24 h. DMSO was used as a negative control. Subsequently, the luciferase activity was measured and normalized to β-galactosidase activity. The results were expressed as the fold induction (over the activity of the negative control)

Recently, it has been reported that many signaling pathways, such as classic Wnt and LIF signaling pathway including JAK/STAT3, PI3K/Akt were involved in regulation the stemness of stem cells [35]. Therefore, activation of Wnt, JAK/STAT3 and PI3K/Akt signaling pathways were investigated in P19 cells treated with MDLS. As shown in Fig. 8A–C, the phosphorylation levels of STAT3 and the entry of β-Catenin into the nucleus were increased in P19 cells after treatment with MDLS for 15 min, whereas the phosphorylation levels of Akt did not change significantly, indicating that MDLS could activate both JAK/STAT3 and classic Wnt signaling pathways. Considering that MDLS could increase the expression of Oct4 (Fig. 2B and C), we hypothesized that MDLS promoted the expression of Oct4 through activating the JAK/STAT3 and classic Wnt signaling pathways. Then, Ruxolitinib [36] and Salinomycin [37] were employed to block the JAK/STAT3 and classic Wnt signaling pathways respectively. As expected, the results in Fig. 8D showed that Ruxolitinib significantly reserved the up-regulated expression levels of Oct4 caused by MDLS. Similar results were clearly observed in P19 cells treated by Salinomycin (Fig. 8E). In addition, the effect of Ruxolitinib and Salinomycin on the promoter of Oct4 was also tested and the results indicated that both Ruxolitinib and Salinomycin successfully reserved the increased levels of Oct4 caused by MDLS (Fig. 8F). Furthermore, we investigated whether there were synergistic effects between MDLS and LIF on these pathways, and we observed that combination of MDLS and LIF had a superior effect on JAK/STAT3 signaling than that of either drug alone. However, it seemed that there was no indication of synergistic effects on Wnt signaling (Additional file 1: Fig. S1). Taken together, these findings reinforced the hypothesis that MDLS promoted the expression of Oct4 at least partially through the JAK/STAT3 and classic Wnt signaling pathway.

## Discussion

Self-renewal and pluripotency are key properties of stem cells which enable them to function in different stages of development. However, these properties of stem cells would get lost in the process of expansion in vitro. Therefore, it is urgent to develop effective approach to expand stem cells. Recently, the role of small-molecule compounds in regulating stem cell fate raised lots of concerns [1, 18, 19, 38]. Oct4 has been well known to be critical in establishment and maintenance of pluripotency of ESCs [39, 40]. Hence, the current study aimed to screen small-molecule compounds targeting Oct4 for maintaining pluripotency.

Here, we screened 480 small-molecule compounds by two luciferase reporters, which targeted the promoter and 3’-UTR of Oct4 respectively. Transcriptional regulation is the key mechanism that controls gene expression and cell fate. By directly affecting binding of DNA-binding proteins and proteins recruited by them to promoter or indirectly regulating related signaling pathways, drugs possess the ability to modulate the transcription pattern of genes in response to changing signals [41]. Thus, we might find useful potential drugs that can regulate gene expression and cell function by testing the promoter activity. In addition to transcription or translation regulation, 3′ UTRs of mRNAs are best known to regulate mRNA-based processes, including mRNA localization, mRNA stability, and translation. In addition to the role in miRNA-mediated regulation, the miRNA-independent functions of 3′ UTRs were also very important. Actually, most functions of 3′ UTRs were mediated by RNA-binding proteins (RBPs). Most importantly, it was reported that AU-rich elements were predominantly found in 3′ UTRs of a certain class of genes that encodes cytokines, lymphokines, growth factors, and oncogenes [42, 43]. AU-rich elements were critical regulatory elements that enable fast and highly dynamic regulation of protein production with a sharp on and off switch by binding to RBPs [44]. Therefore, 3’-UTR luciferase reporters of Oct4 are good model reporter for drug screening.

Furthermore, the effects of the compound screened on the expression of Oct4 at the mRNA and protein levels was elucidated by RT-qPCR and west blotting assay. Based on the cell cytotoxicity analysis, MDLS was selected for further investigation (Fig. 3). MDLS, 2-methyl-3-piperidinopyrazine derivative, had the ability of inhibiting monoamine oxidase (MAO) [45, 46] and showed therapeutic anti-depressant activity [47]. MDLS potentiated convulsions induced by tryptamine, antagonised reserpine induced hypothermia, and hyperthermia induced by dopa [46]. Besides, peripheral administration of MDLS resulted in hypotension due to ganglionic blockade [48]. On the other hand, MDLS reinforced monosynaptic spinal reflexes caused by stimulation of brain stem reticular for mation [49]. It was reported that MDLS injected intracerebroventricularly induced a biphasic pressor response consisting of initial and delayed phases [50]. However, little is known about the effect of MDLS on stem cells. Therefore, it is worth determining whether MDLS acts as a potential small molecule for maintaining self-renewal and pluripotency of stem cells.

The results (Fig. 3A–C) showed that MDLS could effectively promote the clone formation of P19 cells. In order to exclude this possibility that clone formation was caused by cell proliferation, the effect of MDLS on the proliferation of P19 cells was detected. The results showed that MDLS had no effect on cell proliferation and it was effective in reinforcing pluripotency in P19 cells (Figs. 4 and 5). Pluripotent stem cells have the potential of differentiating into cells of all three germ layers [51]. While, the histological results validated that MDLS-treated teratomas could differentiated into many typical gut-like tissues, pancreatic acini-like tissues (endoderm) and mesenchymal tissues (mesoderm) in our study (Fig. 5E). Consistent with this, the expression levels of AFP and cTnT, as markers for endoderm and mesoderm respectively, remarkably up-regulated in MDLS-induced teratomas (Fig. 5F). However, the expression levels of β-tubulin (the marker of ectoderm) also increased in MDLS-induced teratomas (Fig. 5F). These data indicated that MDLS-treated teratomas may also differentiated into ectoderm, but we didn’t observe it by the histological analysis.

The core transcription factors Oct4, Nanog and Sox2 formed a positive-feedback Loop and the expression levels of them needed to be kept at appropriate levels to maintain pluripotency [52]. For example, Oct4 overexpression induced differentiation toward mesoderm and endoderm lineages; the increase in the levels of Sox2 overexpression promoted neuroectodermal specification [53, 54]. The balanced pluripotent state suggested that Oct4 and Sox2 mutually regulated each other in lineage specification [55]. The results (Figs. 4C–E and 7A) showed that MDLS up-regulated the expression levels of Oct4 and Sox2, avoiding exceedingly high levels of Oct4. It is noteworthy that the impact of MDLS on maintaining self-renewal and pluripotency of stem cells has not yet been reported.

Mechanistically, we found that MDLS could increase the phosphorylation of STAT3 and nuclear translocation of β-Catenin (Fig. 8A and C). We also revealed that blocking of both JAK/STAT3 and classic Wnt signaling pathways reversed the MDLS-induced up-regulation of Oct4 (Fig. 8D and E), suggesting that JAK/STAT3 and classic Wnt signaling pathways were required for the MDLS-induced expression of Oct4. However, whether other signals also participated in the MDLS-induced up-regulation of Oct4 was still unclear. Moreover, how MDLS regulated the expression of Oct4 though JAK/STAT3 and classic Wnt signaling pathways was not known and needed further exploration.

In summary, we have firstly identified a small molecule, modaline sulfate (MDLS), enhanced Oct4 and maintained the self-renewal and pluripotency of P19 and mES cells. In addition, the results revealed that MDLS regulated the expression of Oct4 through JAK/STAT3 and classic Wnt signaling pathways. Although the detailed mechanisms require further investigation, MDLS maybe a novel small molecule for maintaining self-renewal and pluripotency of stem cells in vitro and vivo.

## Supplementary Information

**Additional file 1**: **Figure S1**. The synergistic effects between MDLS and LIF on p-STAT3 and Wnt signaling pathways. (A) Left: the phosphorylation levels of Akt were detected by western blot analysis in P19 cells. Right: quantitative analysis of Western blotting data by image J soft-ware. Levels of these proteins were normalized to the corresponding GAPDH band expressed as relative fold changes in comparison to control samples. Values correspond to the mean ±S.E. of three independent experiments. (B) The synergistic effects between MDLS and LIF on Wnt signaling was investigated. Left: the levels of β-Catenin in the nucleus were detected by western blot analysis in P19 cells. Right: quantitative analysis of Western blotting data by image J soft-ware. Levels of these proteins were normalized to the corresponding Histone3 band expressed as relative fold changes in comparison to control samples. Values correspond to the mean ±S.E. of three independent experiments.

## Data Availability

The datasets used and/or analyzed during the current study are available from the corresponding author on reasonable request.

## Abbreviations

P19 cells: Mouse embryonic carcinoma cells line
HEK-293T cells: Human embryonic kidney cell line
OG2-mES: Mouse embryonic stem cells
Oct4: Octamer-binding transcription factor-4
MDLS: Modaline sulfate
AP: Alkaline phosphatase
Sox2: SRY-related high-mobility-group -box protein-2
EG: Embryonic germ
FACS: Fluorescence-activated cell sorting
PFA: Paraformaldehyde
