# Supplementary material for: Modaline sulfate promotes Oct4 expression and maintains self-renewal and pluripotency of stem cells through JAK/STAT3 and Wnt signaling pathways
Source: Cell Biosci. 2021 Aug 4;11:156. doi: 10.1186/s13578-021-00669-3 (PMC8336387; doi:10.1186/s13578-021-00669-3)
Supplement: Supplementary file 1 — Additional file 1: Figure S1. The synergistic effects between MDLS and LIF on p-STAT3 and Wnt signaling pathways. (A) Left: the phosphorylation levels of Akt were detected by western blot analysis in P19 cells. Right: quantitative analysis of Western blotting data by image J soft-ware. Levels of these proteins were normalized to the corresponding GAPDH band expressed as relative fold changes in comparison to control samples. Values correspond to the mean ±S.E. of three independent experiments. (B) The synergistic effects between MDLS and LIF on Wnt signaling was investigated. Left: the levels of β-Catenin in the nucleus were detected by western blot analysis in P19 cells. Right: quantitative analysis of Western blotting data by image J soft-ware. Levels of these proteins were normalized to the corresponding Histone3 band expressed as relative fold changes in comparison to control samples. Values correspond to the mean ±S.E. of three independent experiments. [file 13578_2021_669_MOESM1_ESM.docx]

Caption：The synergistic effects between MDLS and LIF on p-STAT3 and Wnt signaling pathways

Figure S1


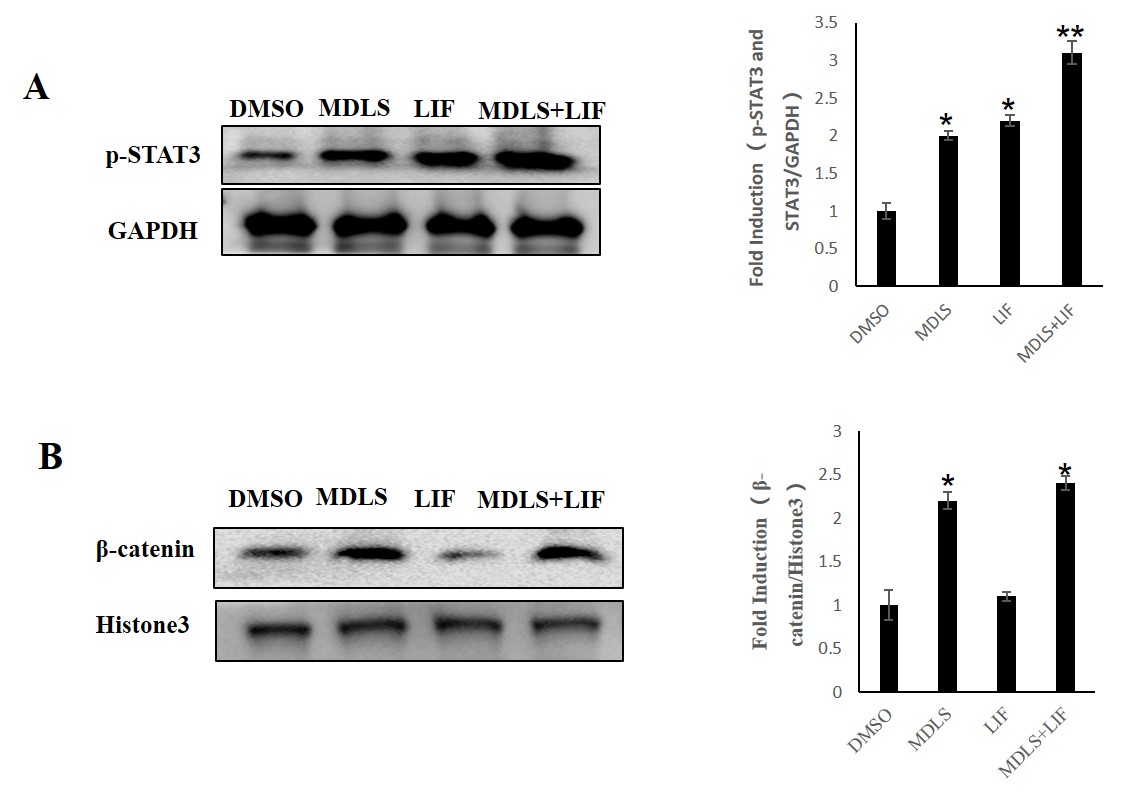


The synergistic effects between MDLS and LIF on p-STAT3 and Wnt signaling pathways

(A) Left: the phosphorylation levels of Akt were detected by western blot analysis in P19 cells. Right: quantitative analysis of Western blotting data by image J soft-ware. Levels of these proteins were normalized to the corresponding GAPDH band expressed as relative fold changes in comparison to control samples. Values correspond to the mean ±S.E. of three independent experiments.

(B) The synergistic effects between MDLS and LIF on Wnt signaling was investigated.

Left: the levels of β-Catenin in the nucleus were detected by western blot analysis in P19 cells. Right: quantitative analysis of Western blotting data by image J soft-ware. Levels of these proteins were normalized to the corresponding Histone3 band expressed as relative fold changes in comparison to control samples. Values correspond to the mean ±S.E. of three independent experiments.
